# Pulmonary and cardiovascular responses to repeated ozone exposure during exercise in adults with exercise‐induced bronchoconstriction

**DOI:** 10.14814/phy2.70977

**Published:** 2026-06-26

**Authors:** Patric Emerson Oliveira Gonçalves, Bennett Stothers, Andy Hung, André Casanova Silveira, Owen Drake Harris, Tessa van de Kerkhof, Nadine Borduas‐Dedekind, Andrew William Sheel, Michael Stephen Koehle

**Affiliations:** ^1^ School of Kinesiology, Faculty of Education The University of British Columbia Vancouver British Columbia Canada; ^2^ School of Physical Education and Sport University of São Paulo São Paulo Brazil; ^3^ Radboud Universiteit Nijmegen The Netherlands; ^4^ Department of Chemistry, Faculty of Science The University of British Columbia Vancouver British Columbia Canada; ^5^ Division of Sport & Exercise Medicine, Faculty of Medicine The University of British Columbia Vancouver British Columbia Canada

**Keywords:** air pollution, asthma, exercise‐induced bronchoconstriction, spirometry

## Abstract

We tested the hypothesis that exercising while breathing real‐world ozone (O_3_) concentrations impairs pulmonary and cardiovascular function in individuals with exercise‐induced bronchoconstriction (EIB) and that repeated exposures induce acclimation. Participants completed consecutive 30‐min cycling sessions breathing room air (RA) and 170 ppb O_3_. A mixed‐effects model and ANOVA were used. Sixteen individuals (50% male) with mild–moderate EIB completed the study. O_3_ exposure produced pulmonary impairment on Day 2 in forced expiratory volume in 1 s (Estimate = −5.1, 95% CI: −8.5 to −1.7, *p* = 0.003), forced vital capacity (Estimate = −4.0, 95% CI: −7.1 to −0.9, *p* = 0.008), and forced expiratory flow in the midportion of vital capacity (Estimate = −6.7, 95% CI: −12.7 to −0.7, p = 0.008). Minute ventilation was lower on Days 3 and 4 in O_3_, *p* = 0.007 and *p* = 0.001. Diastolic blood pressure was lowest on Day 2 under O_3_, 58 mmHg (95% CI: 52, 64) versus RA, 63 mmHg (95% CI: 58, 69), and a significant overall lower HR in O_3_ was found across all visits, *p* = 0.032. These findings demonstrate a significant difference in pulmonary and cardiovascular responses to O_3_ compared to RA, but no statistical evidence of acclimation.

## 1 INTRODUCTION

Air pollution is a major risk factor for pulmonary and cardiovascular disease, and is the greatest environmental contributor to mortality, accounting for 7 million premature deaths worldwide (WHO, 2021). Greater ventilation during exercise, along with the tendency to bypass nasal filtration at higher intensities, can increase the effective dose of inhaled pollutants and therefore the potential for harmful health effects (Giles & Koehle, 2014; Hung et al., 2023; Sandford et al., 2021). Ground‐level ozone (O_3_) is a gaseous air pollutant created secondarily when ultraviolet light acts on volatile organic compounds and oxides of nitrogen, forming O_3_ (Trainer et al., 2000). Ozone exposure can induce bronchoconstriction, reduce oxygen uptake during exercise, alter ventilatory response, and impair athletic performance (Giles & Koehle, 2014; Harris et al., 2024; Hung et al., 2022). However, studies have demonstrated a potential ability of individuals to acclimate to O_3_, leading to an attenuation of its pulmonary impacts (Folinsbee et al., 1980; Gong et al., 1997).

Proposed mechanisms underlying acclimation to repeated air pollutant exposure include a reduction in inflammation—marked by a decreased count of neutrophils and IL‐6 in bronchoalveolar lavage fluid—and desensitization of irritant receptors (Christian et al., 1998; Giles & Koehle, 2014). Exercise‐induced bronchoconstriction (EIB) is a condition with up to 50% prevalence in athletes and up to 90% in those with asthma as described by Parsons & Mastronarde. The severity of EIB correlates with eosinophilic inflammation, which can be aggravated by O_3_ exposures, directly impairing lung function and increasing airways responsiveness (Folinsbee et al., 1994; Parsons & Mastronarde, 2005). However, studies on O_3_ acclimation have been largely focused on healthy populations and pulmonary outcomes, leaving its cardiovascular impacts poorly understood. When triggered by air pollution, respiratory responses can impact autonomic regulation, resulting in changes in heart rate variability and arterial stiffness (Koch et al., 2020; Lundbäck et al., 2009).

Thus, understanding how individuals with EIB respond to repeated exercise sessions in realistic O_3_ level exposure is important both for understanding the phenomenon and also for counseling active individuals with EIB. The present study therefore sought to determine whether repeated exposures to a controlled level of O_3_ while exercising could mitigate the impairment in pulmonary and cardiovascular function in individuals with EIB. We hypothesized that ozone exposure during exercise would lead to pulmonary and cardiovascular function decrements and ventilatory response changes that would be mitigated with subsequent exposures.

## 2 MATERIALS AND METHODS

### 2.1 Ethical approval

This study was registered under the clinicaltrials.gov #NCT05105529. The University of British Columbia's Clinical Research Ethics Board approved this study under the registration #H21‐01183. This study was conducted in accordance with the Declaration of Helsinki. All participants provided written informed consent.

### 2.2 Participants and recruitment

Participants were recruited through flyers on public advertising around the university common areas, a recruiting website at the School of Kinesiology, the University of British Columbia Psychology graduate student council's newsletter, craigslist, and word‐of‐mouth.

We included adults with EIB, between the age of 18–65 years old, that were able to communicate in English and give informed consent, who were physically active non‐smokers, and able to safely perform maximal‐intensity exercise testing. Potential participants who reached out to our lab and had been told by their family physicians that they had EIB, or those who suspected that they had EIB and met the abovementioned criteria were invited to perform a eucapnic voluntary hyperventilation test (EVH). If they experienced a 10% or greater decrease in forced expiratory volume in the first second (FEV_1_) following the test, they were invited to participate in the study. We did not perform clinical diagnostic tests of asthma; rather, participants were asked to report if they had received an asthma diagnosis from their family physician (Table 1).

**TABLE 1:** Baseline characteristics. Data are shown in means (±SD) or occurrence (%).

	All (*N* = 16)	Females (*N* = 8)	Males (*N* = 8)
Age, years	29 (± 11.5)	28 (± 10)	30 (± 12)
BMI, kg/m^2^	23.4 (± 2.3)	23.3 (± 2.6)	24.8 (± 3)
Asthma diagnosis, *N* (%)	8 (50%)	5 (62.5%)	3 (37.5%)
FEV_1_ drop in the EVH test, %	18.7 (± 10.2)	17.7 (±11.5)	19.6 (± 9.5)
FEV_1_/FVC ratio	0.78 (± 0.1)	0.77 (± 0.1)	0.76 (± 0.1)

*Note*: Age is shown in years.

Abbreviations: BMI, body mass index; EVH, eucapnic voluntary hyperventilation test; FEF_25‐75_, forced expiratory flow in the midportion of the FVC maneuver in liters per second; FEV_1_, forced expiratory volume in the first second; FVC, forced vital capacity.

We did not include individuals that had any conditions that could interfere with or preclude cycling, were diagnosed with cardiorespiratory diseases other than asthma/EIB, or vascular diseases that might impact performance. For the same reasons, we also did not include those on continuous asthma therapy, such as inhaled corticosteroids or asthma biologics. We also did not include those having experienced respiratory symptoms or acute conditions in the month prior to data collection, individuals that were pregnant or potentially pregnant, those on antioxidants, vitamins E or C, estrogen therapy, antihypertensives, or beta‐blockers.

### 2.3 Experimental design and protocol

This experiment used a double‐blind study protocol following the CONSORT statement for randomized crossover trials (Dwan et al., 2019). All participants were exposed to two environmental conditions: breathing 170 ppb O_3_ or breathing room air (RA), while performing submaximal heavy‐intensity exercise on a stationary bike for 30 min. The O_3_ level of 170 ppb was selected to mimic potential real‐world scenarios that individuals could encounter in large urban centres. Participants were not given a period to warm up before the 30 min exercise and were constantly asked to keep intensity at the prescribed power output.

Participants finished their second week of tests at no more than one and a half months apart from week one of exposures. In this study, 87.5% of the participants finished both study arms within the two‐week period following their start date. We did not control for exposure to other environmental triggers, such as dust, pollen, or other air pollutants. However, the laboratory is in an environment with no direct exposure to motor vehicles or other major sources of air pollutants and was temperature‐controlled at 23°C with a relative humidity of 40%.

**FIGURE 1:**
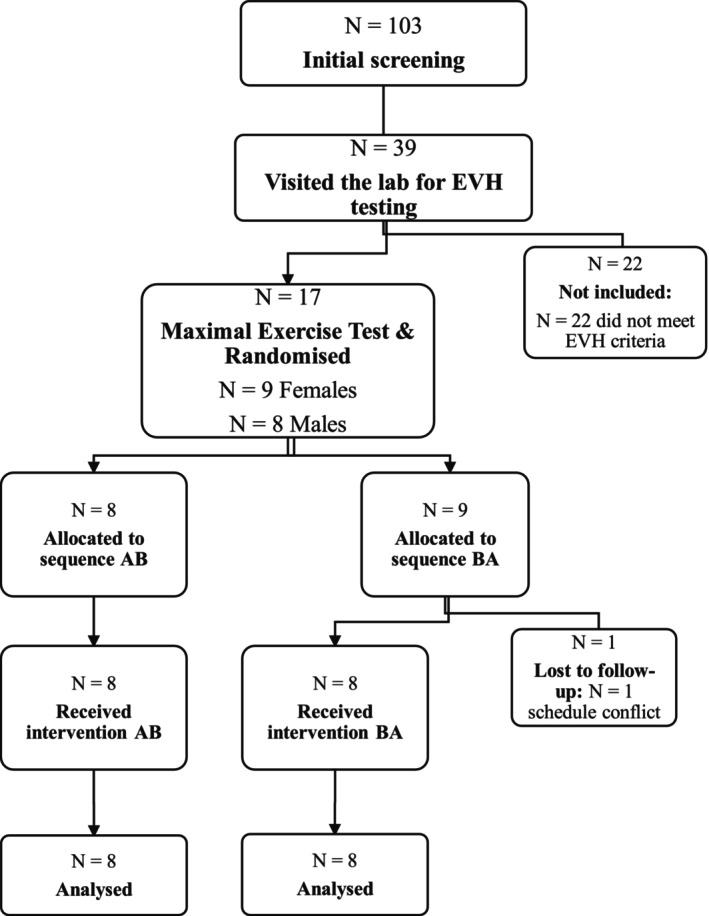
Participant flow diagram. Exercise‐induced bronchoconstriction (EIB). AB, allocation sequence where participants started on ozone exposure; BA, allocation sequence where participants started on room air exposure.

During the screening visit, the EVH test was chosen to minimize impact of an exercise challenge prior to a cardiopulmonary exercise testing (Koch et al., 2018). During the EVH, potential participants were asked to breathe at a target minute ventilation of 30 times their baseline FEV_1_. Individuals then performed a maximal graded exercise test using a ramp protocol on a Velotron DynaFit Pro cycle ergometer (Racemate Inc., Seattle, WA, USA) in room air (ParvoMedics TrueOne® 2400 metabolic measurement system). After a self‐paced warm‐up, the protocol started at 30 W and for males the workload increased 1 W every 2 s while for females 1 W increase every 3 s. Test was terminated upon volitional fatigue, or if they could not maintain 60 rpm. Participants cycled for 30 min at 60% of the peak wattage achieved in the maximal test. Given that past studies have largely chosen intermittent exposure protocols, this protocol was chosen to mimic a realistic exercise bout and based on previous studies that found adaptation at similar exercise intensity range (Folinsbee et al., 1994; Foxcroft & Adams, 1986; Stothers et al., 2024). Following the screening visit, participants who demonstrated EIB with the EVH test were randomized to one of the allocation sequences, Figure 1.

After the first five visits in one environmental condition, participants completed a washout period of at least 48 h (Fakhrzadeh et al., 2002; Folinsbee & Hazucha, 2000) and returned to the laboratory for another five visits under the alternative condition.

Participants were tested at random phases of their menstrual cycle. Data were collected at approximately the same time each day. No participant reported having taken a short‐acting beta‐2 agonist at least 8 h prior to each study visit.

### 2.4 Ozone deliver

The 170 ppb O_3_ was produced with a corona discharge generator (ACT‐5000 Ozonetech Mellifiq, Sweden) and averaged 170.6 ± 1.8 ppb. Mixed room air and generated O_3_ were delivered into a sealed environmental chamber (3 × 3 × 2 m). Participants cycled on the Velotron located outside of the chamber while breathing the mixture air through a 3 m length of wide‐bore, polyethylene EVA copolymer tubing connected to a mouthpiece with a non‐rebreathing valve. Nose clips were used to maintain ventilation through the mouth solely. Neither the participants nor the researchers were inside the environmental chamber with all tests being conducted outside of the chamber. The chamber sealing and the study protocol were methodically planned to prevent the unblinding of participants and researchers by any potential O_3_ smell. Room air (i.e., RA) was delivered with the same setup and natural O_3_ concentrations in that environment averaged 7.2 ± 6.4 ppb.

### 2.5 Blinding

To maintain investigator blinding, a trained research assistant operated the O_3_ generator, concealing the exposure condition during all study visits, including the RA‐only visits. Finally, participants were only told the exposure protocol they were assigned to by a research assistant not directly involved with data collection and only after their study visits were fully completed in both conditions.

### 2.6 Pulmonary response

Spirometry was assessed with triplicate maneuvers in accordance with the American Thoracic Society Guidelines (Graham et al., 2019). Spirometry was done before exercise, immediately after exercise, and 30 min post‐exercise using the ParvoMedics OUS‐SPIRO (Salt Lake City, UT, USA), which was calibrated prior to each participant. The 30‐min mark post‐exercise spirometry was chosen to assess for any significant residual effect of environmental condition to safely discharge participant.

### 2.7 Respiratory symptoms

Participants were asked to report respiratory symptom intensity by choosing a number on a 0–10 modified Borg scale before exercise, at the 30‐min exercise mark, and 30 min post‐exercise (Borg, 1982). Symptoms were chosen based on other studies that investigated the same level of O_3_ air pollution (Lippmann, 1989).

### 2.8 Ventilatory response

Respiratory rate (*f*_B_) in breaths per minute (bpm), tidal volume (V_T_) in liters, minute ventilation (V_E_) in liters per minute (L/min) were taken using LabChart version 8 for Windows (PowerLab 16/35, 2014, ADInstruments Pty Ltd., Australia). Ventilatory and cardiovascular responses were taken at the steady‐state 20‐min mark to minimize end‐of‐session changes in cycling pattern.

### 2.9 Cardiovascular response

Heart rate was measured with a Polar heart rate monitor (Polar Electro Oy, N2965) placed around the chest connected to the PowerLab. Systolic and diastolic blood pressure (SBP and DBP) were recorded using an automated blood pressure cuff (A&D Engineering Inc., Mississauga, ON, CA) at rest to minimize the risk of abnormally high resting blood pressure (Muntner et al., 2019). For the exercise blood pressure, a manual aneroid sphygmomanometer was chosen and, along with HR, SBP, and DBP, were taken at the 20‐min mark of exercise.

### 2.10 Statistical analysis

A sample size of 16 subjects was required to provide 80% power to detect a significant effect at the 0.05 alpha level. The calculation was based on a previous study that investigated changes to FEV_1_ in a similar 5‐day O_3_ exposure protocol (Folinsbee et al., 1994).

A block randomization scheme was generated prior to data collection using random.org. A sequence of eight blocks of two was generated. This allowed a balanced sample size, as well as allocation concealment.

Changes in FEV_1_, FVC, FEF_25‐75_ after each exposure were expressed as a percentage change from the pre‐exposure spirometry from Day 1, considered as the baseline spirometry. This method was selected to quantify potential cumulative or acclimatory effects of repeated exposures on lung function across the week. To test for differences between conditions at baseline, independent *t*‐tests were performed for between‐groups characteristics at baseline.

Pooled (main) effects and interactions were assessed using a two‐way ANOVA (Type III test) approach was applied to a linear mixed‐effects model. Type III analysis of variance was used to evaluate the overall contribution of each fixed effect (i.e., environmental conditions, study visits, and condition × study visit interaction), and participants were specified as random‐effects. When applicable, post hoc pairwise comparisons with Tukey's test were performed when main effects were significant. Pairwise comparisons of estimate of means are depicted as delta (∆): O_3_ – RA followed by *p* value.

All models were fitted using RStudio Version 2024.12.1 (Integrated Development for R, Rstudio, PBC, Boston, MA) with appropriate packages for estimated marginal contrasts (e.g., lme4, Lmertest and emmeans).

## 3 RESULTS

A total of 103 people contacted our team, 39 had their EVH test performed, of which 17 met the EVH fall index criterion and were invited to participate, Figure 1. Sixteen healthy adults with EIB were included, of which 8 were females and 8 were males (Table 1). The study dropout rate was 6%, which represents one participant who had a scheduling conflict and was not able to complete all sessions before the data collection period had ended. Thus, the results shown in this section represent data for the 16 participants that completed the two sets of five consecutive visits in their originally assigned allocation sequence. Our participants considered themselves physically active and half had been diagnosed with asthma, while all participants had mild‐to‐moderate EIB on the pre‐screening EVH test. On the bike test, average VO_2peak_ was 36.5 mL/kg/min ± 9.8 for all participants, with females average being 32.6 mL/kg/min ± 7.1 and males 40.4 mL/kg/min ± 11. The maximal peak power output achieved during the test was 247 W ± 92.3 for the group, while females achieved 187 W ± 61 and males 297 W ± 69. Other baseline characteristics are summarized in Table 1. No statistically significant difference was found for baseline demographic characteristics between those starting on O_3_ or RA.

Data were analyzed according to the original group to which participants were assigned at the commencement of the study. That said, there were no changes in the randomization sequence and allocation.

### 3.1 Pulmonary function

**FIGURE 2:**
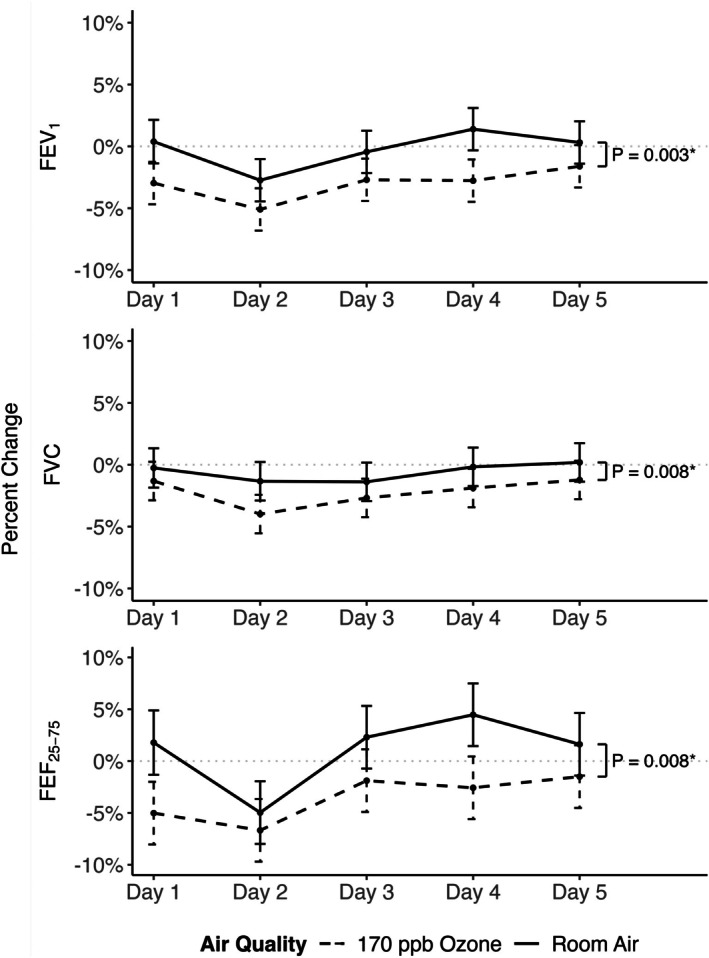
Lung function estimated means percent change across study visits by environmental condition. *p*‐value is for pooled main effect of Ozone versus Room Air. *FEV*_*1*_, forced expiratory volume in the first second; *FEF*_25‐75_, forced expiratory flow in the midportion of the FVC maneuver in liters per second. Error bars represent standard error from the mean. *p < 0.05.

Model‐predicted changes in pulmonary function are shown in Figure 2. There was a significantly lower FEV_1_ in the pooled effect in O_3_ environment condition (*p* = 0.003) compared to that observed in RA, Table 2. Pairwise analysis of FEV_1_ showed no statistically significant differences between environmental conditions. On Day 1, the difference of the estimate means showed a *∆ =* −3.68 L with a *p* = 0.22; on Day 2, the *∆ =* −0.97 L, *p* = 0.99; followed by a *∆ =* −0.92 L, p = 0.99 on Day 3; while on Day 4 the *∆ =* −1.75 L, *p* = 0.96; and on Day 5 the *∆ =* −2.05 L, *p* = 0.89.

**TABLE 2:** Model‐predicted percent change in pulmonary function. Data are shown as estimated marginal means (95% CI: Low, high).

		Day 1	Day 2	Day 3	Day 4	Day 5	*p*‐value
FEV_1_ (L)	O_3_	−3.0 (−6.4, 0.4)	−5.1 (−8.5, −1.7)	−2.7 (−6.1, 0.7)	−2.8 (−6.2, 0.6)	−1.6 (−5.0, 1.8)	0.003*
	RA	0.4 (−3.1, 3.9)	−2.7 (−6.1, 0.7)	−0.4 (−3.8, 2.9)	1.4 (−2.0, 4.8)	0.3 (−3.1, 3.7)	
FVC (L)	O_3_	−1.3 (−4.4, 1.8)	−4.0 (−7.1, −0.9)	−2.7 (−5.8, 0.4)	−1.9 (−5.0, 1.2)	−1.2 (−4.3, 1.9)	0.008*
	RA	−0.3 (−3.4, 2.9)	−1.3 (−4.4, 1.8)	−1.4 (−4.5, 1.7)	−0.2 (−3.3, 2.9)	0.2 (−2.9, 3.3)	
FEF_25‐75_ (L/s)	O_3_	−5.0 (−11.0, 1.0)	−6.7 (−12.7, −0.7)	−1.9 (−7.9, 4.1)	−2.6 (−8.6, 3.4)	−1.5 (−7.5, 4.5)	0.008*
	RA	1.8 (−4.4, 7.9)	−5.0 (−11.0, 1.0)	2.3 (−3.7, 8.3)	4.5 (−1.5, 10.5)	1.6 (−4.4, 7.6)	

Abbreviations: FEF_25‐75_, forced expiratory flow in the midportion of the FVC maneuver in liters per second; FEV_1_, forced expiratory volume in the first second; FVC, forced vital capacity; O_3_, ozone exposure; RA, room air exposure.

* Indicates statistically significant difference, *p* < 0.05, in the analysis of pooled effect of environment condition.

The pooled effect of O_3_ on FVC showed a *p* = 0.008. Pairwise analysis of FVC showed no statistically significant differences between environmental conditions. On Day 1, the difference of the estimate means was *∆ =* −1.39 L with a *p* = 0.97; on Day 2, *∆ =* −0.86 L, p = 0.99; followed by a *∆ =* −1.35 L, p = 0.97 on Day 3, while on Day 4, the *∆ =* −0.99 L, *p* = 0.99; and on Day 5, the *∆ =* −2.39 L, *p* = 0.55.

And the overall pooled effect of O_3_ on FEF_25‐75_ was also statistically significant, p = 0.008. Pairwise analysis of FEF_25‐75_ showed no statistically significant differences between environmental conditions. On Day 1, the difference of the estimate means showed a *∆ =* 0.07 L/s with a *p* = 0.75; on Day 2, *∆ =* 0.02 L/s, *p* = 1; followed by a *∆ =* 0.04 L/s, *p* = 0.98 on Day 3; while on Day 4, *∆ =* 0.07 L/s, *p* = 0.69; and on Day 5, *∆ =* 0.03 L/s, *p* = 0.99.

No statistical difference was observed on the effect of the study visit for pulmonary function, for which FEV_1_ depicted a *p* = 0.19, while FVC had a *p* = 0.406, and FEF_25‐75_ showed a *p* = 0.09. There were no significant effects of interaction between environmental condition and study visit observed for FEV_1_, *p* = 0.63, for FVC, *p* = 0.89, nor for FEF_25‐75_, *p* = 0.82.

After 30 min of exercise, there was no significant pooled effect of the environmental condition on pulmonary function, Table 3. There were also no significant main effects of study visits nor interaction effects in the 30‐min post‐exercise spirometry.

**TABLE 3:** Raw values of spirometry pre, immediately after (peak) and 30 min post exercise (post).

	Day 1		Day 2		Day 3		Day 4		Day 5	
	O_3_	RA	O_3_	RA	O_3_	RA	O_3_	RA	O_3_	RA
FEV_1_ (L)										
Pre	3.7 (0.8)	3.7 (0.7)	3.5 (0.7)	3.6 (0.8)	3.6 (0.7)	3.6 (0.8)	3.6 (0.7)	3.7 (0.7)	3.6 (0.7)	3.6 (0.7)
Peak	3.6 (0.7)	3.7 (0.7)	3.5 (0.7)	3.6 (0.8)	3.6 (0.7)	3.7 (0.8)	3.6 (0.7)	3.7 (0.7)	3.6 (0.6)	3.7 (0.7)
Post	3.6 (0.8)	3.7 (0.8)	3.6 (0.8)	3.6 (0.9)	3.6 (0.7)	3.8 (0.8)	3.6 (0.7)	3.8 (0.8)	3.7 (0.7)	3.8 (0.7)
FVC (L)										
Pre	4.8 (1.0)	4.8 (1.0)	4.6 (1.0)	4.7 (1.1)	4.7 (1.0)	4.7 (1.0)	4.7 (1.0)	4.7 (1.0)	4.7 (0.9)	4.7 (1.0)
Peak	4.7 (1.0)	4.8 (1.0)	4.6 (1.0)	4.7 (1.1)	4.7 (1.0)	4.7 (1.0)	4.7 (1.0)	4.8 (1.0)	4.7 (0.9)	4.8 (0.9)
Post	4.7 (1.0)	5.0 (1.0)	4.7 (1.1)	4.9 (1.2)	4.8 (1.1)	4.9 (1.0)	4.7 (1.0)	4.9 (1.0)	4.8 (1.0)	4.9 (0.9)
FEF_25‐75_ (L/s)										
Pre	3.3 (1.1)	3.2 (0.9)	3.1 (1.1)	3.1 (1.2)	3.1 (1.0)	3.2 (1.1)	3.2 (1.1)	3.3 (1.0)	3.3 (1.2)	3.2 (1.1)
Peak	3.2 (1.1)	3.3 (1.0)	3.1 (1.0)	3.1 (1.2)	3.2 (1.0)	3.3 (1.0)	3.3 (1.3)	3.4 (1.1)	3.3 (1.1)	3.3 (1.1)
Post	3.2 (1.3)	3.1 (1.1)	3.2 (1.0)	2.9 (1.2)	3.1 (1.1)	3.2 (1.0)	3.2 (1.3)	3.2 (1.1)	3.3 (1.3)	3.2 (1.1)

*Note*: Data are shown in means (±SD).

Abbreviations: FEF_25‐75_, forced expiratory flow in the midportion of the FVC maneuver in liters per second; FEV_1_, forced expiratory volume in the first second; FVC, forced vital capacity; O_3_, ozone exposure; RA, room air exposure.

### 3.2 Respiratory symptoms

**FIGURE 3:**
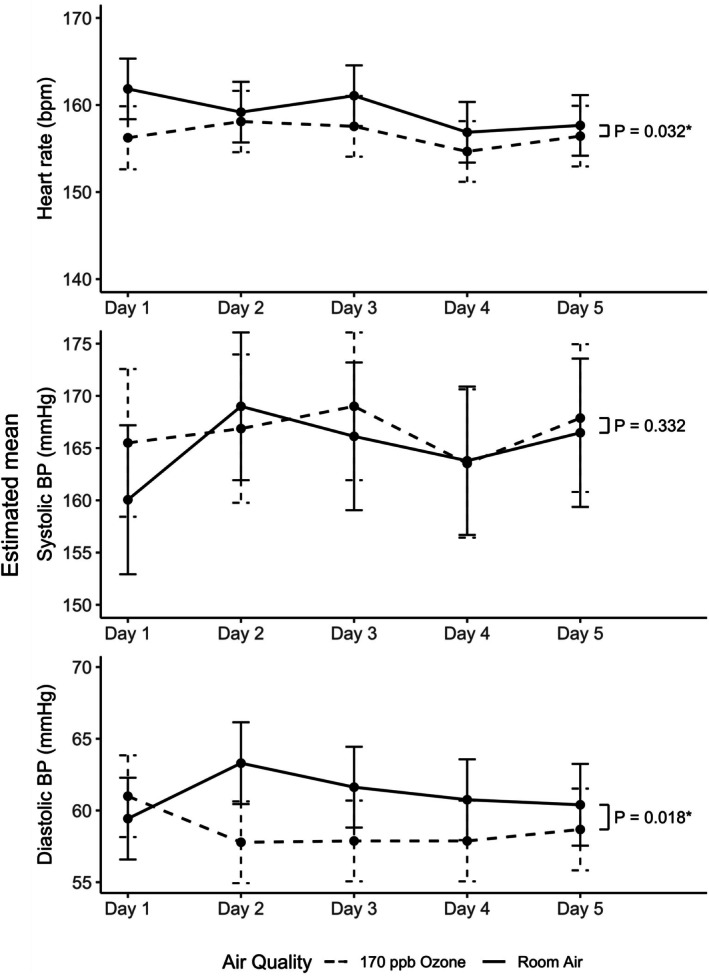
Symptoms reported on a scale from 0 to 10. Boxplot are represented by the median and the interquartile range (IQR: 25th–75th percentile). Whiskers extend to the most extreme data points within 1.5 × IQR, and dots represent outliers.

No statistically significant difference was found in the linear mixed‐effect model for dyspnea and symptoms, Figure 3.

### 3.3 Ventilatory response

Exercise V_E_, V_T_, or *f*_B_ showed no statistical difference for the pooled effect of environment condition, with *p* = 0.080, *p* = 0.746, and *p* = 0.084, respectively, Table 4.

**TABLE 4:** Estimated marginal means of pooled effects of ventilatory response at 20 min of exercise and 95% confidence intervals.

		Day 1	Day 2	Day 3	Day 4	Day 5	*p*‐value
V_E_ (L/min)	O_3_	72.5 (62.8, 82.3)	68.1 (58.5, 77.8)	65.2 (55.5, 74.9)	63 (53.4, 72.7)	69.4 (59.7, 79.1)	*p* = 0.08
	RA	72.5 (62.8, 82.2)	70.4 (60.7, 80.1)	66.3 (56.5, 76.0)	68.9 (59.3, 78.6)	70.7 (61.0, 80.4)	
V_T_ (L/breath)	O_3_	2.1 (1.8, 2.4)	2.1 (1.8, 2.3)	1.9 (1.7, 2.2)	1.9 (1.6, 2.2)	2.1 (1.8, 2.3)	*p* = 0.746
	RA	2 (1.8, 2.3)	2 (1.8, 2.3)	1.9 (1.7, 2.3)	2 (1.7, 2.3)	2.1 (1.8, 2.4)	
*f*_B_ (bpm)	O_3_	35 (31, 39)	34 (30, 38)	34 (30, 38)	34 (30, 38)	34 (31, 38)	*p* = 0.084
	RA	36 (32, 40)	35 (31, 39)	35 (31, 38)	35 (31, 39)	35 (31, 39)	

*Note*: Statistically significant difference was set at *p* < 0.05.

Abbreviations: *f*_B_, breathing frequency in breaths per minute (bpm); O_3_, 170 ppb ozone exposure; RA, room air exposure; V_E_, minute ventilation in liters per minute; V_T_, tidal volume, in liters per breath.

Compared to Day 1 in O_3_, V_E_ was significantly lower on Day 3 in the same environmental condition, ∆ = −7.34 L/min, *p* = 0.007. It was also statistically significantly lower on Day 4 of O_3_ compared to Day 1 in O_3_, ∆ = −9.5 L/min, *p* = 0.001.

On Day 4, V_T_ was significantly lower in O_3_ compared to Day 1 on the same environmental condition, ∆‐0.23 L/breath, *p* = 0.014. There were no interaction effects of environment condition and study visits on V_E_, *p* = 0.558 or V_T_, *p* = 0.612.

There were no statistical effects of study visit on *f*_B_, *p* = 0.663, nor any interaction effect (*p* = 0.982).

### 3.4 Cardiovascular response

Analysis of the cardiovascular response revealed a significant pooled effect of environmental condition with lower HR found after O_3_ exposure compared to RA (*p* = 0.032). Pairwise analysis of HR showed no statistically significant differences between environmental conditions. On Day 1, the difference of the estimate means was ∆ = −6 bpm with a p = 0.66; on Day 2 ∆ = −1 bpm, *p* = 1; followed by a ∆ = −4 bpm, *p* = 0.96 on Day 3, while on Day 4, a ∆ = −3 bpm, *p* = 0.99; and on Day 5 a ∆ = −1 bpm, *p* = 1.

**FIGURE 4:**
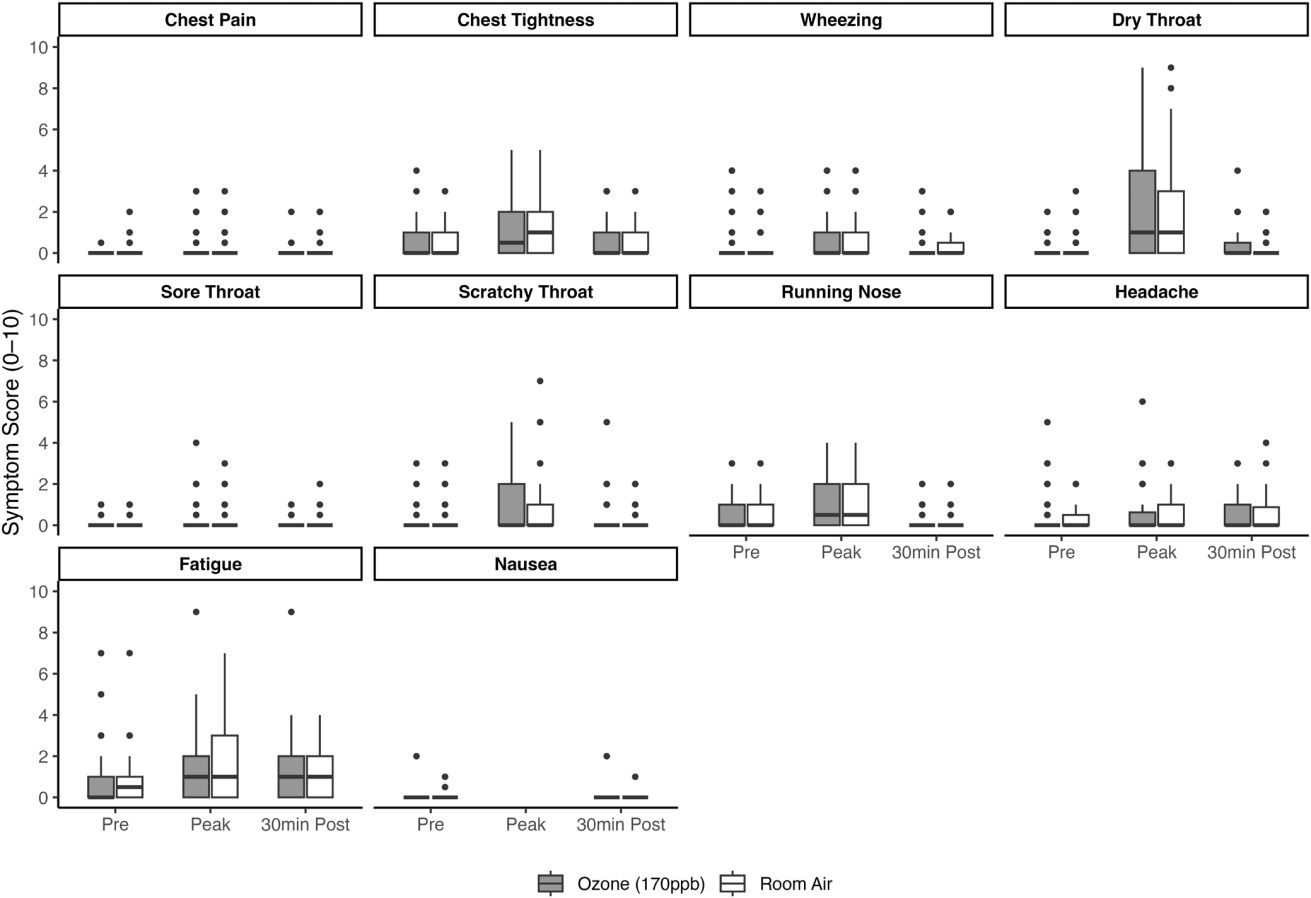
Cardiovascular response estimated means across study visits by environmental condition. Heart rate is shown in beats per minute (bpm), systolic and diastolic blood pressure in millimeters of mercury (mmHg). Error bars represent standard error from the mean. * indicates statistically significant difference between conditions (*p* < 0.05).

There was no statistically significant impact of study visits on HR, *p* = 0.328, Figure 4.

No interaction effect of environment condition and study visit affected HR, *p* = 0.787.

There were no effects on SBP from the pooled environmental condition exposure, *p* = 0.332, from the repeated study visits, *p* = 0.094, nor from the interaction between study visit and environmental condition, *p* = 0.332, Figure 4.

Overall, DBP was significantly lower in O_3_ than RA at 20 min into exercise through Days 2–5, with a pooled environmental significant difference of *p* = 0.018. On Day 1, the difference of the estimate means was ∆ = 2 mmHg, *p* = 0.999, on Day 2, the *∆ =* −6 mmHg, *p* = 0.353, while on Day 3 the ∆ = −4 mmHg, *p* = 0.811, on Day 4, *∆ =* −3 mmHg, *p* = 0.957, and on Day 5, the *∆ =* −2 mmHg, *p* = 0.999.

No statistically significant effects on DBP were observed from different study visits, *p* = 0.943, nor was any significant interaction effect found, *p* = 0.274.

## 4 DISCUSSION

### 4.1 Main findings

We found significant effects of O_3_ exposure on estimated means in the mixed‐effect model on pulmonary (FEV_1_, FVC, FEF_25‐75_), ventilatory (V_T_ and V_E_), and cardiovascular responses (HR and DBP), with no difference in reported symptoms. We found no statistically significant evidence of acclimation to 170 ppb O_3_ in our studied population, contrary to prior exercise protocols in which healthy individuals or those with asthma showed an attenuated bronchoconstrictive response following repeated exposures to 200–400 ppb O_3_ (Gong et al., 1997; Jörres et al., 2000).

### 4.2 Pulmonary function

The magnitude and time course of the pulmonary function decrements in response to O_3_ in the current study are worth putting into context.

Regardless of the trend in adaptation, there are several potential causes for the inability to demonstrate a statistically significant acclimation effect in the present study: O_3_ concentration, exercise intensity, blinding and randomization. In the current study, we chose 170 ppb for the inspired O_3_ concentration for two reasons. Since this is the first study investigating acclimation in a clinical (EIB) population, we used a lower concentration for a greater margin of safety. Secondly, we wanted to use an O_3_ concentration that was representative of the upper limit of a realistic environmental exposure. By contrast, studies that show a potential for acclimation to acute O_3_ exposures have been in healthy populations and have used higher O_3_ concentrations not representative of ambient exposure, typically 350–500 ppb (Folinsbee et al., 1980; Foxcroft & Adams, 1986; Horvath et al., 1981). For example, a study in healthy males by Folinsbee et al. showed a decrease in the pulmonary impairment on the third day of exposure to 350 and 500 ppb of ozone but not to 200 ppb (Folinsbee et al., 1980). Exposing our participants to 170 ppb, we found a smaller initial drop in FEV_1_ on Day 1 of 3%, followed by 5.1% on Day 2, with a partial recovery to 1.6% below baseline on Day 5, but with no statistically significant evidence of acclimation. Similarly, a study that exposed people with exercise‐induced asthma to 120 ppb O_3_ showed a significant decrease in FEV_1_ but no statistically significant evidence of acclimation following the first exposure (Linn et al., 1994). Further evidence for a relationship between concentration and acclimation comes from post‐exercise ozone responses. Following each exposure session in the current study, there were no residual pulmonary function impacts by 30 min post‐exercise. In contrast, a study performed in healthy individuals that exercised for 60 min in 350 ppb O_3_ showed impairments in pulmonary function responses up to 18 h after exposure (Folinsbee & Hazucha, 2000), which suggests a dose‐dependent relationship between O_3_ concentration and its impacts on pulmonary response. Therefore, the smaller and more transient lung function recovery from immediately post‐exercise impact in the present study indicates that the concentration of O_3_ used in our protocol may not have sufficed to cause persisting impairments across study visits.

Exercise intensity may be another explanation for the lack of acclimation effect. The current study used a higher exercise intensity compared to previous studies. Historically, studies have typically used a minute ventilation target (e.g. 40 L/min) instead of a relative workload target. These prior studies have therefore used exercise intensities in the lower end of the moderate intensity domain, whereas in the current study, participants exercised at 60% of the peak power reached during their VO_2peak_ test, which would be classified as heavy intensity exercise. In previous work in our lab, we have noted a greater O_3_ effect in these lower intensity efforts than in maximal efforts, indicating that the higher intensity exercise might mask the O_3_ effect on lung function (Harris et al., 2024). Similarly, with other pollutants like diesel exhaust, we have found that the adverse effects of pollution were more pronounced during moderate than heavy exercise (Giles et al., 2018). Indeed, there is some evidence that an increase in minute ventilation (as observed during more intense exercise) seems to mitigate bronchoconstriction caused my methacholine. This has been demonstrated with hyperpnea caused by both hypoxia and carbon dioxide (Pellegrino et al., 2009; Torchio et al., 2022). The mechanism of the attenuated bronchoconstrictive response is believed to be related to an increase in stretching of airway smooth muscle brought about by the greater ventilation. This mechanism may also be responsible for a decrease in the bronchoconstrictive effect of ozone present in higher‐intensity exercise.

Finally, unlike earlier studies, the current study used both randomization and double‐blinding (of researcher and participant) protocol in order to reduce the potential for bias, especially in subjective responses and in effort‐dependent responses (such as spirometry). It is possible that by reducing the potential for these biases, spurious acclimation‐like phenomena from participants expecting a given response to air pollution were prevented.

### 4.3 Ventilatory pattern

We also hypothesized that ventilatory pattern could be affected by O_3_ exposure in people with EIB. Studies in both animal models and humans showed that in addition to bronchoconstriction caused by O_3_, rapid and shallow breathing patterns were also found (Foxcroft & Adams, 1986; Schelegle et al., 2001). One theory for this phenomenon suggests that in healthy rats, C‐fiber stimulation by 1000 ppb O_3_ could function as a defense mechanism to mitigate conducting airway damage by augmenting *f*_B_ and decreasing V_T_ (Schelegle et al., 2001). We did find V_T_ and V_E_ to be significantly lower in O_3_, but only on Day 4, with a trend towards less shallow breathing on Day 5. However, the day‐specific reduction in VE appears to be driven primarily by a decrease in V_T_ rather than a *f*_B_‐V_T_ response as expected to follow the C‐fiber stimulation mechanism pathway. This suggests that the modest O_3_ dose in our protocol was likely not enough to trigger that rapid‐shallow breathing pattern or that people with EIB show a different response than those without EIB. As with the acclimation effects, perhaps the combination of increased exercise intensity and decreased concentration compared to previous research explains the lack of ventilatory pattern change in this group.

### 4.4 Cardiovascular response

The present study demonstrated a non‐statistically significant decrease in HR with its lowest in O_3_ of 6 bpm and DBP, with a nadir of 6 mmHg on Day 2. A previous study conducted in healthy subjects also found a similar decrease in HR of 7 bpm during greater exposure to 370 ppb of ozone (Folinsbee et al., 1975), but not during ozone concentrations of 500 and 750 ppb. Another study from our group showed similar HR tendency in healthy highly trained athletes exposed to the same O_3_ concentration at a much higher exercise intensity in time‐to‐exhaustion trials (Harris et al., 2024). During heavy intensity exercise in O_3_, that study found a 2 bpm lower HR compared to RA although not statistically significant (*p* = 0.052). Although this mild bradycardia mechanism is still unclear in the EIB population, the cardiovascular response to O_3_ may reflect altered autonomic balance and a potential non‐linear dose–response. Furthermore, electrocardiography would be relevant to clarify changes in ventricular repolarization and depolarization in future investigations, given that exercising in O_3_ has been shown to increase QT interval duration, which at certain levels can have significant impacts such as arrhythmias or lethal cardiac events (Devlin et al., 2012).

### 4.5 Respiratory symptoms

We hypothesized that respiratory symptoms would increase following air pollution exposure; however, they were not significantly different between the two studied environmental conditions. Although dry throat, chest tightness, and wheezing were reported more frequently immediately after exercise, most of these symptoms were present similarly in the two conditions. Therefore, we believe that the pre‐existing EIB condition and the low O_3_ dose could be responsible for the lack of contrast in symptoms between the exposures in the two environments. There is some conflicting evidence of symptom acclimation with repeated exposure to ozone in asthmatics (Jörres et al., 2000) and healthy individuals (Foxcroft & Adams, 1986). In the Foxcroft and Adams study, the reduction in symptoms occurred over multiple exposures with a simultaneous reduction in ozone dose. Thus, it is unclear whether the symptom reduction was due to reduced dose or acclimation (Foxcroft & Adams, 1986).

### 4.6 Limitations

Of the participants, although 50% had previously been clinically diagnosed with chronic asthma, their condition was mild enough for them to forgo their medication for the duration of this study. On the other hand, participants in our study showed a considerable heterogeneity in airway responsiveness to the EVH test, and ranged from 10% to more marked 25% post‐EVH decline in FEV_1_. For future studies, considering different asthma/EIB phenotypes could bring more clarity to the underlying mechanisms of pulmonary response to real‐world levels of O_3_. It is also important to highlight that although our participants reported being physically active, some of them found the 30‐min protocol quite challenging. Thus, the exercise protocol could have been a co‐intervention (i.e., a training stimulus) such that over each 5‐day study arm, participants derived a training benefit that might have masked an acclimation effect. However, with the blinding and randomization, this training effect would have at least been balanced across study arms. Another limitation of this study was that we did not control for inhaled dose of O_3_ or potential environmental pollutants and for menstrual cycle phases at time of each study visit.

Our more realistic O_3_ concentration and exercise protocol are strengths of the study, in terms of generalisability, but have likely led to more modest O_3_ and EIB effects due to a relatively lower inhaled dose and exercise intensity. Considering EIB response can be load dependent (Carlsen et al., 2000), future research that increases inhaled dose through higher exercise intensity or longer duration exercise (instead of higher concentration) might observe greater pulmonary and cardiovascular consequences of the O_3_, and might lead to more evidence of acclimation, but with less realistic ambient O_3_ concentrations. Finally, future studies should directly investigate responses in individuals with and without EIB to elicit whether the magnitude of cardiopulmonary response impacts performance, providing more supportive evidence to decision‐makers such as healthcare providers, coaches, and trainers.

## 5 CONCLUSION

In individuals with EIB, exposure to 170 ppb O_3_ during 30 min of heavy exercise impaired pulmonary function, decreased HR and DBP, but there was no statistically significant acclimation observed with repeated exposures. Although those changes were modest, this study highlights that even at real‐world O_3_ levels, individuals with multisystem conditions or those in physically demanding activities could be exposed to such effects.

## AUTHOR CONTRIBUTIONS

**Patric Emerson Oliveira Gonçalves:** Conceptualization; data curation; formal analysis; investigation; methodology; project administration; software; validation; visualization. **Bennett Stothers:** Conceptualization; data curation; formal analysis; investigation; methodology; validation; visualization. **Andy Hung:** Conceptualization; data curation; formal analysis; investigation; methodology. **André Casanova Silveira:** Formal analysis; investigation; methodology. **Owen Drake Harris:** Conceptualization; formal analysis; investigation; methodology; validation; visualization. **Tessa van de Kerkhof:** Conceptualization; data curation; investigation; methodology; project administration; validation; visualization. **Nadine Borduas‐Dedekind:** Conceptualization; data curation; formal analysis; funding acquisition; investigation; methodology; project administration; resources; software; supervision; validation; visualization. **Andrew William Sheel:** Conceptualization; data curation; formal analysis; funding acquisition; investigation; methodology; project administration; resources; software; supervision; validation; visualization. **Michael Stephen Koehle:** Conceptualization; data curation; formal analysis; funding acquisition; investigation; methodology; project administration; resources; software; supervision; validation; visualization.

## FUNDING INFORMATION

This study was funded by the Government of Canada | Natural Sciences and Engineering Research Council of Canada (NSERC) under RGPIN‐2017‐04519 and RTI‐2021‐00091, and University of British Columbia (F20‐06008).

## CONFLICT OF INTEREST STATEMENT

The authors declare no conflicts of interest.

## ETHICS STATEMENT

The University of British Columbia's Clinical Research Ethics Board approved this study under the # H21‐01183.

## Data Availability

Data is available upon request and following appropriate ethical approval and data transfer agreements.
